# Experimental Chronic Prostatitis/Chronic Pelvic Pain Syndrome Increases Anxiety-Like Behavior: The Role of Brain Oxidative Stress, Serum Corticosterone, and Hippocampal Parvalbumin-Positive Interneurons

**DOI:** 10.1155/2021/6687493

**Published:** 2021-03-02

**Authors:** Nikola Šutulović, Željko Grubač, Sonja Šuvakov, Djurdja Jerotić, Nela Puškaš, Djuro Macut, Aleksandra Rašić-Marković, Tatjana Simić, Olivera Stanojlović, Dragan Hrnčić

**Affiliations:** ^1^Institute of Medical Physiology “Richard Burian”, Belgrade University Faculty of Medicine, 11000 Belgrade, Serbia; ^2^Institute of Clinical and Medical Biochemistry, Belgrade University Faculty of Medicine, 11000 Belgrade, Serbia; ^3^Institute of Histology and Embryology “Aleksandar Đ. Kostić”, Belgrade University Faculty of Medicine, 11000 Belgrade, Serbia; ^4^Clinic of Endocrinology, Diabetes and Metabolic Disease, CCS, Belgrade University Faculty of Medicine, 11000 Belgrade, Serbia

## Abstract

Mechanisms of the brain-related comorbidities in chronic prostatitis/chronic pelvic pain syndrome (CP/CPPS) are still largely unknown, although CP/CPPS is one of the major urological problems in middle-aged men, while these neuropsychological incapacities considerably diminish life quality. The objectives of this study were to assess behavioral patterns in rats with CP/CPPS and to determine whether these patterns depend on alterations in the brain oxidative stress, corticosterone, and hippocampal parvalbumin-positive (PV+) interneurons. Adult male *Wistar albino* rats from CP/CPPS (intraprostatic injection of 3% *λ*-carrageenan, day 0) and sham (0.9% NaCl) groups were subjected to pain and anxiety-like behavior tests (days 2, 3, and 7). Afterwards, rats were sacrificed and biochemical and immunohistochemical analyses were performed. Scrotal allodynia and prostatitis were proven in CP/CPPS, but not in sham rats. Ethological tests (open field, elevated plus maze, and light/dark tests) revealed significantly increased anxiety-like behavior in rats with CP/CPPS comparing to their sham-operated mates starting from day 3, and there were significant intercorrelations among parameters of these tests. Increased oxidative stress in the hippocampus, thalamus, and cerebral cortex, as well as increased serum corticosterone levels and decreased number of hippocampal PV+ neurons, was shown in CP/CPPS rats, compared to sham rats. Increased anxiety-like behavior in CP/CPPS rats was significantly correlated with these brain biochemical and hippocampal immunohistochemical alterations. Therefore, the potential mechanisms of observed behavioral alterations in CP/CPPS rats could be the result of an interplay between increased brain oxidative stress, elevated serum corticosterone level, and loss of hippocampal PV+ interneurons.

## 1. Introduction

Chronic prostatitis/chronic pelvic pain syndrome (CP/CPPS) represents a clinical entity whose dominant symptoms are related to pain or discomfort located in the pelvic region [1]. The prevalence of CP/CPPS is very high with 35–50% of men in all age groups reported to be affected by these symptoms during their lifetime [2].

In addition to chronic pelvic pain, CP/CPPS usually causes urinary problems and sexual dysfunction accompanied by psychosocial disorders [3]. These psychosocial issues could, in turn, worsen the CP/CPPS-related dysfunctions in a positive feedback loop manner. Therefore, the syndrome is rarely discussed, kept in secret, and followed by stigma, although its impact on life quality is significant [4].

CP/CPPS patients display higher stress levels, and 62% of them show anxiety symptoms [5]. Hypothalamic-pituitary-adrenal (HPA) axis dysfunction is suspected to be one of the main mechanisms connecting CP/CPPS with anxiety in these patients [6]. Moreover, pain itself is associated with intense recruitment of the HPA axis and corticosterone release, interfering profoundly in the animal behavior [7]. Clinically relevant anxiety has a higher prevalence among patients with chronic pain. Although clinical and epidemiological studies are convincing regarding the relationship between CP/CPPS and anxiety, there is the limited number of *in vivo* experimental studies regarding this relationship [8–10], so potential pathophysiological mechanisms underpinning this relationship are still largely unknown. The experimental model of CP/CPPS induced by intraprostatic injection of *λ*-carrageenan has been shown as one of the valuable models for exploring its etiopathology as well as comorbidities [11].

Nowadays, most experts believe that CP/CPPS is a noninfectious syndrome [12] since no evidence of bacteria in prostate tissue has been found in affected individuals [13]. Tissue injury or stressful events trigger chronic peripheral inflammation or nerve injury [14] resulting in high production of inflammatory mediators and neurotransmitters [15] in the prostate. Consequently, it lowers the threshold of primary nociceptive neurons (peripheral sensitization) and leads to the development of inflammatory/neuropathic pain. Several studies indicated the role of oxidative stress in CP/CPPS patients [16–18]. Namely, prostate tissue inflammation is followed by increased reactive oxygen species (ROS) production [19]. However, there are no studies on the role of ROS in the brain-related comorbidities, including anxiety, in CP/CPPS in experimental settings. Also, it is unknown whether oxidative stress can develop in tissues far from an affected organ, as in the brain [20] and its structures which play important roles in anxiety development: the hippocampus, thalamus, or cortex.

The hippocampus plays an important role in the regulation of the HPA axis and also represents a key point in stress, pain response [21], and emotional processes [22]. Dysfunction of local hippocampal GABAergic inhibitory interneuron networks has been shown in many psychiatric illnesses, including anxiety, depression, and posttraumatic stress disorder [23]. Among these interneurons, parvalbumin-positive (PV+) interneurons are highly represented in the hippocampus [21] and provide inhibitory inputs that regulate the synaptic excitation and control the timing of information flow [23]. Recent studies revealed decreased hippocampal PV+ interneurons in chronic stress [24], chronic pain [21], human psychiatric diseases [23], and oxidative stress-driven neuroinflammation [25]. These findings indicate their potential role in anxiety, as well as their vulnerability to chronic stress-evoked signalling and altered redox status. However, the influence of CP/CPPS on hippocampal PV+ interneurons is still unknown, as well as its contribution to CP/CPPS-related bran comorbidities.

Taking into account current considerations, we hypothesized that CP/CPPS is linked to alterations in anxiety-like behavior concomitantly with imbalances in the brain oxidative stress, serum corticosterone, and hippocampal PV+ interneurons. This relationship has not been previously experimentally investigated to the best of our knowledge. Therefore, the objectives of this study were to assess the influence of experimental CP/CPPS on rat anxiety-like behavior, as well as brain oxidative stress level, serum corticosterone level, and the number of hippocampal PV+ interneurons.

## 2. Materials and Methods

### 2.1. Ethical Statement

All experimental procedures were in full compliance with the Directive of the European Parliament and the Council (2010/63/EU) and approved by The Ethical Committee of the University of Belgrade (Permission No. 323-07-01339/2017-05/3).

### 2.2. Animals and Housing

Adult (three-month-old, weighted 250–350 g, total *n* = 12) male *Wistar albino* rats, obtained from the Military Medical Academy breeding laboratory (Belgrade, Serbia), were used in the experiments. The animals were housed in transparent plexiglass cages (55 × 35 × 30 cm) with soft bedding and access to food and water *ad libitum* during the entire experiment. They were kept under controlled ambient conditions (22-24°C, 50 ± 5% relative humidity, 12/12 h light : dark cycle with the light turned on from 08:00 to 20:00 h). Animals were used only once during the experiment. Acclimatization period to the laboratory ambient lasted for 7 days.

### 2.3. Experimental Design and Test Protocol

Based on our previous experiments and literature data [11], rats were randomly divided into control, sham-operated (sham, *n* = 6), and experimental groups with CP/CPPS (CP/CPPS, *n* = 6) on the day of the intraprostatic injection (denoted as day 0 of the experiment). In the experimental group, CP/CPPS was induced by single intraprostatic injection of 3% *λ*-carrageenan (mucopolysaccharide from the cell walls of the red algae). To assess the development of CP/CPPS, we evaluated mechanical pain thresholds in the scrotal skin using an electronic von Frey aesthesiometer (evF). Three days before intraprostatic injection, rats were adapted to the evF aesthesiometer. Mechanical pain thresholds were determined at different time points, 2 days and 1 day before intraprostatic injection, as well as 2, 3, and 7 days upon intraprostatic injection. The animals from both groups (*n* = 6 per group) were subjected to ethological tests to assess the anxiety-related behavior at different time points: 2, 3, and 7 days upon intraprostatic injection.

The standard battery of ethological tests consisted of an open field (OF), elevated plus maze (EPM), and light/dark (L/D) test to which the rats were subjected consecutively (in the following order: OF, EPM, and L/D). The same cohort of CP/CPPS and sham animals that underwent ethological testings (*n* = 6 per group) was sacrificed on the 7^th^ day of the experiment to perform biochemical, histological, and immunohistochemical examinations. It allowed us to correlate experimental output variables from behavioral, biochemical, and immunohistochemical analysis. Serums were collected for corticosterone level determination. Brain structures were isolated for the determination of oxidative stress parameters, as well as the number of hippocampal PV+ interneurons. Prostates were collected for histological examination.

**Figure 1:**
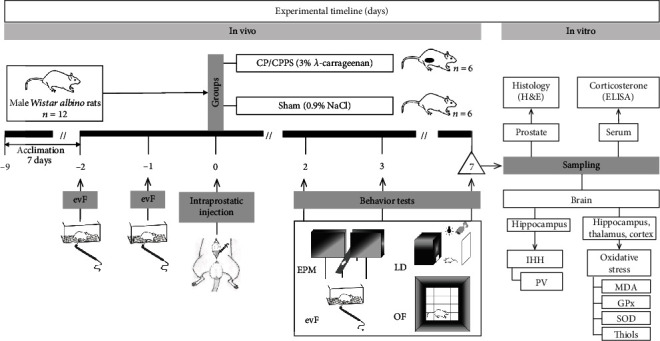
Experimental design. Nine days before surgery (-9), male *Wistar albino* rats (*n* = 12) started to acclimate to laboratory conditions. Depending on the intraprostatic treatment during the operation, rats were randomly divided into two groups: sham (intraprostatic injection of 0.9% NaCl; *n* = 6) and CP/CPPS (intraprostatic injection of 3% *λ*-carrageenan; *n* = 6). Scrotal pain threshold measurement, using an electronic von Frey (evF) esthesiometer, was performed on 2 and 1 day before as well as 2, 3, and 7 days upon intraprostatic injection (0). Behavioral tests (EPM = elevated plus maze test; LD = light/dark test; OF = open field test) were also performed 2, 3, and 7 days upon surgery. On the 7^th^ day upon surgery, immediately after the completion of the tests, rats were sacrificed and prostates were sampled for histology hematoxylin-eosin (H&E) examination; serums were collected for corticosterone measurement by ELISA. Brains were removed from skulls; thereafter, the number of PV+ immunoreactive interneurons in the hippocampus was estimated by immunohistochemistry (IHH), and parameters of oxidative stress (MDA, GPx, SOD, and thiols) were measured in the hippocampus, thalamus, and cortex.

The time course of the experiment is presented schematically in Figure 1.

### 2.4. CP/CPPS Model Induction

Induction of CP/CPPS was performed in the accordance with protocol [11, 26, 27], previously described in detail. Briefly, rats were anaesthetized with pentobarbital sodium (50 mg/kg, i.p.), fixed in a supine position on a heating pad, and the skin of the lower abdomen and scrotum was shaved and disinfected before the surgery. Local anaesthetic (2% lidocaine) was applied to reduce postoperative pain and minimize sensitization of the wound surrounding area. Hereupon, ventral lobes of the prostate gland were exposed through a small midline abdominal wall incision. Sterile suspension of 3% *λ*-carrageenan (Sigma-Aldrich, St. Louis, MO, USA) in a volume of 50 *μ*l was injected in both ventral lobes of the prostate gland (CP/CPPS group). Control animals were treated by intraprostatic injection of the equal volume of sterile 0.9% saline (sham group). After the injection, a 2% lidocaine solution was applied to the wound again and properly closed.

### 2.5. Pelvic Pain Threshold Assessment

An electronic von Frey aesthesiometer (IITC Life Sciences, CA) with rigid filaments was used to assess pelvic pain threshold. Rats were placed in plexiglass cubicles on a wire mesh platform for 30 minutes to adapt. Stimulation was performed when the rat remained quiet with the scrotum resting on the bottom of the cage. Thereafter, evF filament was applied to the scrotal skin with the gradual increase of the pressure, until the animal responded by moving from the original position. The average value of three stimulations was used for analysis. After the measurement of the pain threshold, animals were returned to their respective cages.

### 2.6. Open Field Test (OF)

The open field behavior of rats was monitored by an automated system, fully equipped with infrared sensors (Experimetria Ltd., Budapest, Hungary) and its accompanying software program (Conducta 1.0) as described in detail in our previous study [28]. Briefly, this system registered the horizontal and vertical activity of animals gently placed in the center of the sound-attenuated area (48 cm × 48 cm) with red lighting of 12 lx, surrounded by black walls (height 40 cm). The system recorded the distance and time of ambulatory movement, as well as the number of rearings during rat exploration of the novel environment. The duration of the recording sessions was 15 min. Subsequently, the whole area was divided by the software into 16 squares of which the 4 middle squares were marked as the central area. The time that an animal spent in the central area was measured. The thigmotaxic index was calculated as a ratio between the distance of rat ambulatory movements in the peripheral zones and the total distance of ambulatory movements (%).

### 2.7. Light-Dark Test (L/D)

For the light-dark test, a rat was placed in the center of the light compartment (27 × 27 × 27 cm, all surfaces painted in white) which was connected with the dark compartment (27 × 18 × 27 cm, all surfaces painted in black) by a square aperture (8 × 8 cm, Elunit, Belgrade, Serbia). Rat activity was video monitored during the following 5 min and analyzed offline by an investigator blinded to the treatment. The time that an animal spent in the light compartment of the light-dark test, as well as the number of transitions from light to the dark compartment, was measured as indicators of anxiety-related behavior.

### 2.8. Elevated Plus Maze Test (EPM)

The elevated plus maze apparatus consisted of two open arms (50 × 10 cm) and two enclosed arms (50 × 10 × 40 cm), arranged in such a way that two pairs of identical arms were opposite to each other. Arms emerged from a central platform (10 × 10 cm), and the entire apparatus was raised to a height of 50 cm above floor level. At the beginning of the test, the rat was placed on the central platform facing an open arm. After each 5 min test, the maze was carefully cleaned up to avoid any olfactory trace of the previous animal. Anxiety-related behavior was assessed through the following behavioral parameters forming output variables of this test: time in open arms and the overall number of transitions between opened and closed arms. These parameters are inversely related to the anxiety level in rodents.

### 2.9. Serum Corticosterone Assay

Animals from CP/CPPS and sham groups (*n* = 6 per group) were sacrificed at day 7 of the experiment. Blood samples were collected and centrifuged at 1575 g for 10 min. Serums were collected and frozen at -80°C until assayed for hormone levels. Corticosterone level was determined in sampled serums using commercially available kits by applying the enzyme immunoassay method (ELISA Kit AC-14F1, IDS, United Kingdom) according to the manufacturer's instructions. Minimal detectable concentration was 0.1 ng/ml (detection range: 0.2-400 ng/ml). Duplicate serum aliquots for all hormone analyses were used.

### 2.10. Biochemical Analysis of Oxidative Stress

After sacrificing the rats, brains were carefully removed from the skull. Hereupon, one of the brain hemispheres (alternately left or right) was used for brain oxidative stress assessment, while another one is processed in immunohistochemical analysis. The hippocampus, thalamus, and cortex were isolated from the rat brain and homogenized in 8 volumes of RIPA buffer with added protease inhibitor cocktail to the homogenate. Tubes were centrifuged at 14,000 rpm for 30 minutes at 4°C, and supernatant was collected.

Malondialdehyde (MDA) concentration was measured according to the colourimetric method of Dousset et al. [29], using TBARS (thiobarbituric acid reactive substances). MDA conjugates with TBARS forming red coloured MDA-TBA compound which has a light absorption peak at 532 nm and molar absorption coefficient of 1.56 × 105 l/(mol × cm). Superoxide dismutase (SOD) activity was measured by the method described by Misra and Fridovich [30]. The method is based on the ability of SOD to inhibit autooxidation of epinephrine at alkaline pH (pH 10.2). One unit of SOD activity was defined as the amount of enzyme, which inhibits the oxidation of epinephrine by half. Glutathione peroxidase (GPx) activity was measured by the coupled assay procedure [31]. One unit of enzyme activity is defined as mmol NADPH oxidized/min, assuming 6.22 × 103/l/mol/cm to be the molar absorbency of NADPH at 340 nm. Protein thiol groups (P-SH) were assayed according to the method previously described by Jocelyn [32]. Namely, P-SH reduces DTNB [5,5′-dithiobis-(2-nitrobenzoic acid)] making yellow coloured 5-thio-2-nitrobenzoic acid (TNB). TNB has a molar extinction coefficient of 13.6 × 103 Lml/1 cm at 412 nm wavelength. Nitrotyrosine was assessed by competitive enzyme immunoassay (OxiSelectTM ELISA kits, Cell Biolabs) with a standard curve ranged from 20 nM to 8.0 *μ*M.

### 2.11. Immunohistochemistry Determination of Hippocampal PV+ Neurons

One of the isolated brain hemispheres (alternately left or right) from each CP/CPPS and sham (*n* = 6) rat skull was fixated in a 4% formaldehyde solution in phosphate buffer and embedded in paraffin. Coronal brain sections, 5 *μ*m thick, were dewaxed, rehydrated, and treated with citrate buffer (pH 6.0) in a microwave for antigen retrieval. Endogenous peroxidase activity was blocked with 3% H_2_O_2_, and nonspecific labelling was blocked by normal horse serum. Slices were incubated in primary antibody-mouse monoclonal anti-PV (1 : 1000, Sigma-Aldrich) overnight at room temperature. Labelling was performed using a biotinylated anti-mouse secondary antibody, followed by avidin-biotin-horseradish peroxidase complex (Vector Laboratories). Visualization of the immunoreactive sites was done by 3,3′-diaminobenzidine chromogens (Vector Laboratories). Finally, sections were counterstained with Mayer's hematoxylin and covered. Counting was done on a Leica DM4000 B LED microscope with digital camera Leica DFC295 using the Leica Application Suite (LAS, v4.4.0) software system. Assessments of the hippocampal PV-immunoreactive cells were performed for all animals (*n* = 6 per group). The number of immunoreactive neurons was obtained on the dorsal hippocampus and expressed per 1 mm^2^ of the investigated hippocampal region (CA1, CA2/3, and dentate gyrus-DG). Two independent experimenters who made the counts were blind to the experimental protocols and showed high interrater reliability (Pearson's *r* = 0.95), and the mean value was taken as the final count.

### 2.12. Prostate Histology

Prostates were removed from all rats instantaneously upon the sacrifice, fixed in 10% buffered formalin, dehydrated in ethanol, cleared in xylene, and embedded in paraffin. Five *μ*m thick sections were subjected to routine staining with hematoxylin and eosin (HE) and examined under the Olympus BX41 light microscope with an Olympus C5060A-ADU digital camera.

### 2.13. Data Analysis

The Kolmogorov-Smirnov test was used to test the normal distribution of values. The output variables of behavioral test data, as well as data on biochemical and immunohistochemical analysis, showed normal distribution. Therefore, the results were expressed as means ± standard error (SE) for all parameters. The Student *t*-test was used to evaluate significances of between-group differences (i.e., CP/CPPS vs. sham). The within-group statistical differences were estimated by repeated measurement one-way ANOVA with the Tukey-Kramer LSD post hoc test (i.e., 2, 3, and 7 days). The criteria for the significance of statistical differences were *p* < 0.05, *p* < 0.01, or *p* < 0.001. Pearson correlation coefficient was computed to assess the relationship among MDA levels in different brain tissues, serum corticosterone, and major output variables of anxiety-like behavioral testing, as well as to assess the relationship among the number of PV immunoreactive interneurons in different hippocampal regions (CA1, CA2/3, and DG) and major output variables of anxiety-like behavioral tests.

## 3. Results

### 3.1. Development of Pelvic Pain Syndrome

**Figure 2:**
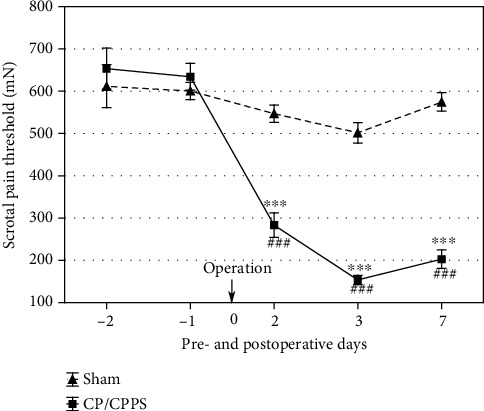
Scrotal pain thresholds in sham and CP/CPPS rats. Scrotal pain thresholds were estimated by evF 2 and 1 day before as well as 2, 3, and 7 days upon operation (0). Values are mean ± SEM. Between-group differences in the scrotal pain threshold were estimated by *t*-test (^∗∗∗^*p* < 0.001, vs. sham, *n* = 6 per group), while within-group differences were estimated by one-way ANOVA with the Tukey-Kramer LSD post hoc test (^###^*p* < 0.001, vs. -1, *n* = 6 per group). For details, see the caption of Figure 1.

Scrotal pain thresholds for mechanical stimuli showed that there were no differences between control and experimental animals (sham vs. CP/CPPS, *p* > 0.05, Figure 2) in basal conditions, i.e., 2 days and 1 day before surgery. Also, there were no differences within the sham group in all measurements taken postsurgery in comparison to basal values. Scrotal pain thresholds in CP/CPPS rats were highly significantly reduced (*p* < 0.001) compared to the thresholds in sham rats on the 2^nd^, 3^rd^, and 7^th^ day upon intraprostatic injection (Figure 2). Also, there is a highly significant reduction (*p* < 0.001) of pain thresholds within the CP/CPPS rats in all measurements taken upon intraprostatic injection in comparison to their basal pain threshold values (Figure 2).

### 3.2. Histological Analysis of Prostates

**Figure 3:**
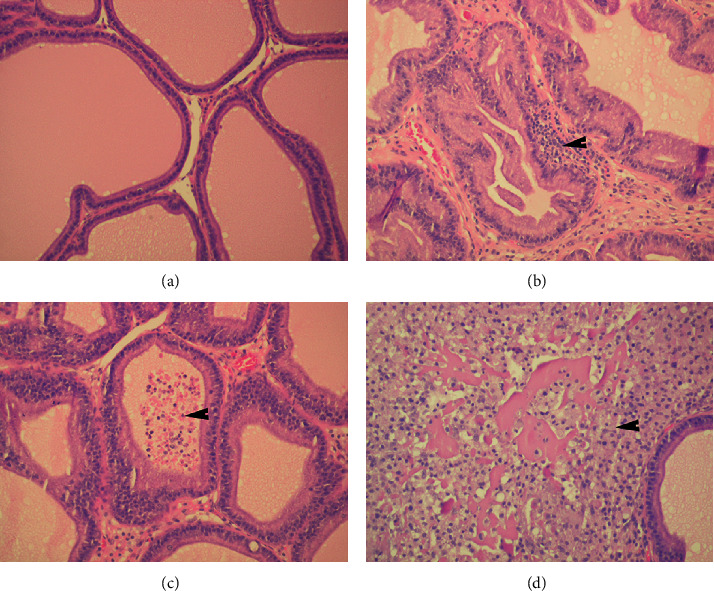
Histopathological features of prostate tissues in sham (a) and CP/CPPS (b–d) rats: preserved histological structure of prostates from the sham group with standard appearance of prostatic glands, interstitium, and well-preserved glandular epithelium (a). Prostates in rats from the CP/CPPS group showed interstitial proliferation with leukocyte infiltration (arrow) (b), cell desquamation and leukocyte infiltration in tubuloalveolar glands (arrow) (c), and interstitial necrosis (arrow) (d). Magnification for all images ×200. For details, see the caption of Figure 1.

Rat prostates injected with 0.9% sterile saline (sham group) showed preserved histological structure with standard appearance of prostatic glands, interstitium, and well-preserved glandular epithelium (Figure 3(a)). On the other hand, intraprostatic injection of 3% *λ*-carrageenan led to the inflammation of the prostate (CP/CPPS group, Figures 3(b)–3(d)). Inflammatory changes were also uniform in all animals in the CP/CPPS group, and they included interstitial proliferation with predominant mononuclear cell infiltration (arrow, Figure 3(b)), cell desquamation and leukocyte infiltration in tubuloalveolar glands (arrow, Figure 3(c)), and interstitial necrosis (arrow, Figure 3(d)). These histological findings are highly suggestive to chronic prostatitis existence.

### 3.3. Anxiety-Like Behavior

#### 3.3.1. Open Field Test

**Figure 4:**
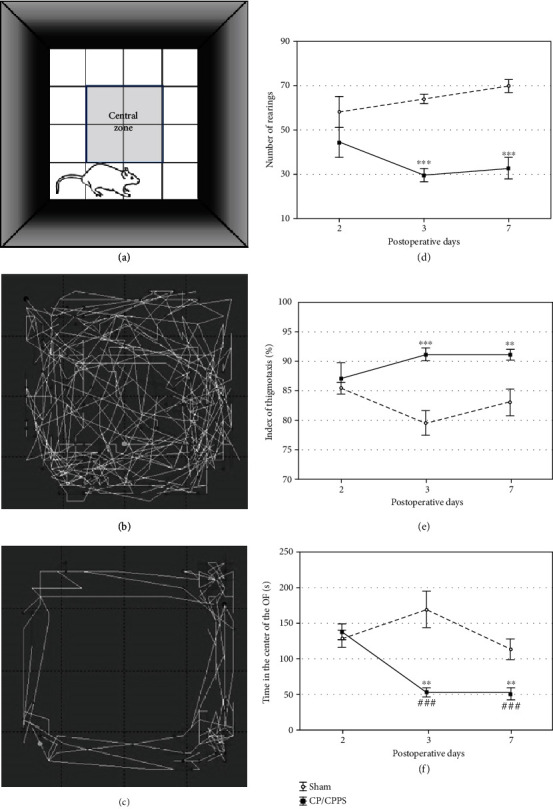
Schematic illustration (a) and representative traces of locomotor activity of animals in the sham group (b) and the CP/CPPS group (c). The number of rearings (d), thigmotaxis index (e), and time spent in the central area (f) in the open field test (OF) registered in sham and CP/CPPS groups. The number of rearings was calculated as the number of times rat has propped on the hind legs. The index of thigmotaxis was defined as a ratio between the distance of ambulatory movement a rat made in the peripheral areas and the total distance of ambulatory movements in the open field test. Values are mean ± SEM. The statistical significance of the difference between the groups was estimated by Student's *t*-test (^∗∗^*p* < 0.01 and ^∗∗∗^*p* < 0.001 vs. sham), while within-group differences were estimated by one-way ANOVA with the Tukey-Kramer LSD post hoc test (^###^*p* < 0.001 vs. 2 d, *n* = 6 per group). For details, see the caption of Figure 1.

Schematic illustration of the OF is shown in Figure 4(a). Analysis of the locomotor activity of animals from the sham and CP/CPPS groups in the OF showed different behavioral patterns, as shown in representative traces of ambulatory movements in the arena of this test (Figures 4(b) and 4(c)).

Further quantitative analysis of anxiety-related output variables 2^nd^ day postoperatively showed no differences between sham and CP/CPPS groups in any of the analyzed parameters (*p* > 0.05, Figures 4(d)–4(f)). On the other hand, differences were revealed between rats who underwent intraprostatic injection of 3% *λ*-carrageenan (CP/CPPS group) and those who underwent intraprostatic injection of 0.9% saline (sham group) on the 3^rd^ as well as 7^th^ day upon the surgery. According to one-way ANOVA with the Tukey-Kramer LSD post hoc test, the number of rearings, an indicator of vertical activity, was significantly decreased in CP/CPPS rats compared to sham rats (*p* < 0.001, Figure 4(d)) on the 3^rd^ and 7^th^ day upon the surgery. The index of thigmotaxis was significantly higher in CP/CPPS rats in comparison with sham rats on the 3^rd^ (*p* < 0.001, Figure 4(e)) and 7^th^ day (*p* < 0.01, Figure 4(e)) upon the surgery. Also, CP/CPPS rats spent significantly less time in the central area of the open field test compared to the sham rats (*p* < 0.01, Figure 4(f)), at the same postoperative days. Besides, there is a highly significant reduction (*p* < 0.001) of the time in the central area of the OF test within the CP/CPPS rats on the 3^rd^ and 7^th^ postoperative day in comparison to the 2^nd^ postoperative day values (Figure 4(f)).

#### 3.3.2. Elevated Plus Maze Test (EPM)

**Figure 5:**
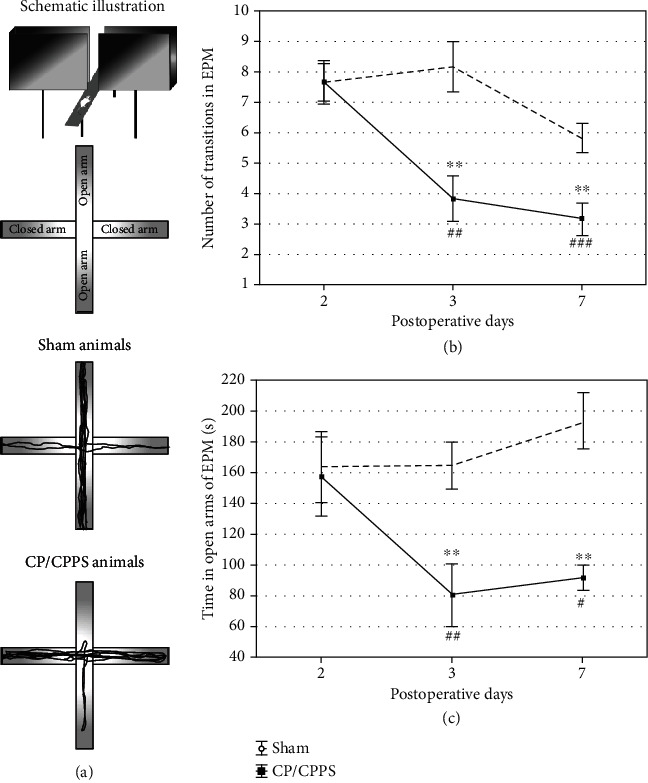
Schematic illustration (a), the number of transitions between the opened and closed arm (b), and the time spent in the open arm (c) in the elevated plus maze test (EPM) observed in sham and CP/CPPS groups. Values are mean ± SEM. The statistical difference between the groups was estimated by Student's *t*-test (^∗∗^*p* < 0.01 vs. sham), while within-group differences were estimated by one-way ANOVA with the Tukey-Kramer LSD post hoc test (^#^*p* < 0.05, ^##^*p* < 0.01, and ^###^*p* < 0.001 vs. 2 d, *n* = 6 per group). For details, see the caption of Figure 1.

Schematic illustration of the EPM is shown in Figure 5(a). No differences were detected between CP/CPPS and sham animals in any of the analyzed parameters derived from EPM (*p* > 0.05, Figures 5(b) and 5(c)) on the 2^nd^ day upon the surgery. However, intraprostatic injection of 3% *λ*-carrageenan (CP/CPPS group) significantly decreased the time animals spent in the open arms during the test compared to control protocol (*p* < 0.01, Figure 5(c)) on the 3^rd^ as well as 7^th^ day upon the surgery. Also, there is a highly significant reduction of the time animals spent in the open arms during the test within the CP/CPPS rats on the 3^rd^ (*p* < 0.01, Figure 5(c)) and 7^th^ day (*p* < 0.05, Figure 5(c)) upon the surgery in comparison to values on the 2^nd^ postoperative day. Further, on the 3^rd^ as well as 7^th^ postoperative day, the number of open/closed arm transitions was significantly decreased in the CP/CPPS group compared to the sham group (*p* < 0.01, Figure 5(b)). Also, there is a highly significant reduction of the number of open/closed arm transitions within the CP/CPPS rats on the 3^rd^ (*p* < 0.01, Figure 5(b)) and 7^th^ postoperative day (*p* < 0.001, Figure 5(b)) in comparison to basal values.

#### 3.3.3. Light/Dark Test (L/D)

**Figure 6:**
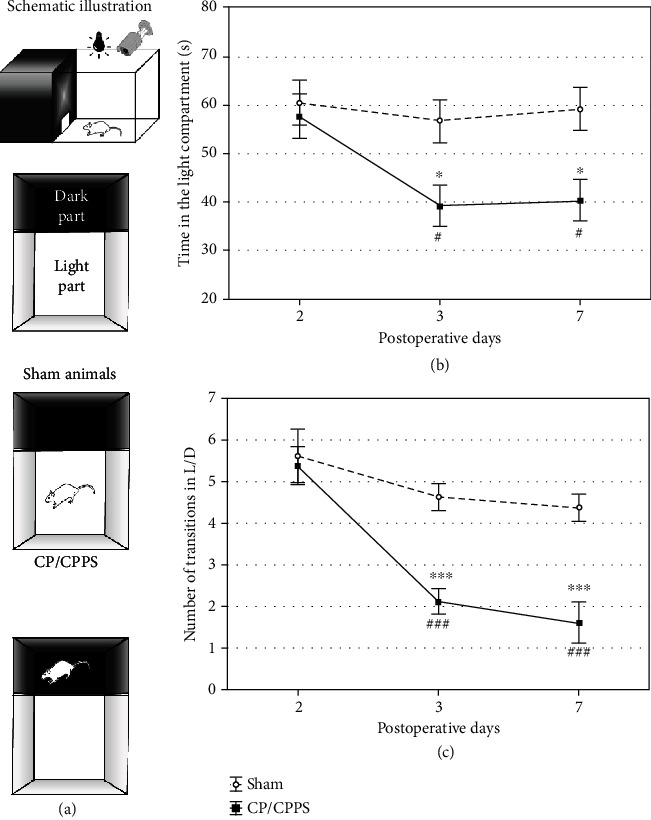
Schematic illustration (a), the time in the light compartment (b), and the number of transitions between the light and the dark compartment (c) in the light/dark test observed in sham and CP/CPPS groups. Values are mean ± SEM. The statistical difference between the groups was estimated by Student's *t*-test (^∗^*p* < 0.05 and ^∗∗∗^*p* < 0.001 vs. sham), while within-group differences were estimated by one-way ANOVA with the Tukey-Kramer LSD post hoc test (^#^*p* < 0.05 and ^###^*p* < 0.001 vs. 2 d, *n* = 6 per group). For details, see the caption of Figure 1.

Schematic illustration of the L/D is shown in Figure 6(a). The time animals spent in the light compartment was significantly shorter in the CP/CPPS group compared to the sham group (*p* < 0.05, Figure 6(b)) on the 3^rd^ as well as 7^th^ day upon the surgery. The same holds for the number of L/D compartment transitions (CP/CPPS vs. sham, *p* < 0.001, Figure 6(c)). These light/dark test parameters were not significantly different between CP/CPPS and sham groups (*p* > 0.05, Figures 6(b) and 6(c)) on the 2^nd^ day upon the surgery. Also, within the group analysis of these parameters showed that CP/CPPS rats spent significantly less time in the light compartment (*p* < 0.05, Figure 6(b)) and made high significantly less light/dark compartment transitions (*p* < 0.001, Figure 6(c)) on the 3^rd^ and 7^th^ postoperative day in comparison to the values on the 2^nd^ postoperative day.

### 3.4. Oxidative Stress in the Brain

**Figure 7:**
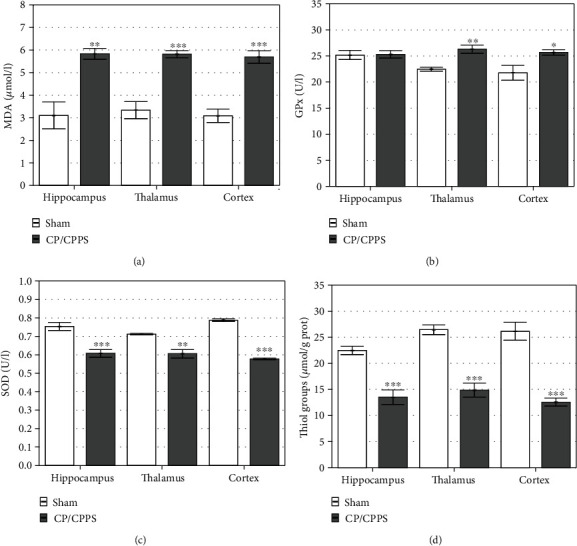
Oxidative stress parameters in the brain structures: malondialdehyde (MDA, a) level, the activity of glutathione peroxidase (GPx, b), the activity of superoxide dismutase (SOD, c), and the level of thiol groups (d) determined in the hippocampus, thalamus, and cerebral cortex in sham and CP/CPPS groups seventh day after intraprostatic injection. Values are mean ± SEM. The statistical difference between the groups was estimated by Student's *t*-test (^∗^*p* < 0.05, ^∗∗^*p* < 0.01, and ^∗∗∗^*p* < 0.001 vs. sham, *n* = 6 per group). For details, see the caption of Figure 1.

Results of biochemical analyses showed a statistically significant increase of the MDA level in the hippocampus (*p* < 0.01), thalamus (*p* < 0.001), and cortex (*p* < 0.001) of CP/CPPS animals compared with sham mates (Figure 7(a)).

Intraprostatic injection of 3% *λ*-carrageenan led to alterations in the activity of antioxidant enzymes in brain structures. Namely, the activity of GPx enzyme was significantly higher in the thalamus (*p* < 0.01) and cortex (*p* < 0.05) of animals from the CP/CPPS group compared with animals from the sham group (Figure 7(b)). On the other hand, there was no statistically significant difference in GPx enzyme activity in the hippocampus of animals from these groups (*p* > 0.05, Figure 7(b)). SOD activity has been uniformly decreased in the hippocampus (*p* < 0.001), thalamus (*p* < 0.01), and cerebral cortex (*p* < 0.001) in the CP/CPPS group compared to the activity of this enzyme in corresponding structures isolated from the sham group (Figure 7(c)). CP/CPPS animals showed a significantly lower thiol group content in all brain structures than sham animals, i.e., hippocampus (*p* < 0.001), thalamus (*p* < 0.001), and cortex (*p* < 0.001; Figure 7(d)).

### 3.5. Serum Corticosterone

**Figure 8:**
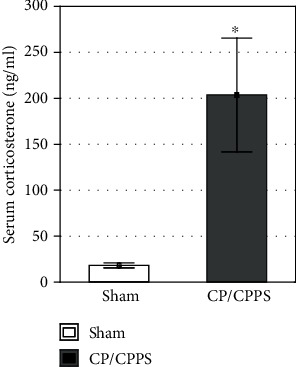
Serum levels of corticosterone in sham and CP/CPPS groups seventh day after intraprostatic injection. Serum concentration levels were determined using the ELISA method. Values are mean ± SEM. The statistical difference between the groups was estimated by Student's *t*-test (^∗^*p* < 0.05 vs. sham, *n* = 6 per group). For details, see the caption of Figure 1.

Serum corticosterone level analysis showed a significant increase in corticosterone level in serum of CP/CPPS animals, compared to the sham group (*p* < 0.05, Figure 8).

### 3.6. Hippocampal PV+ Interneurons

**Figure 9:**
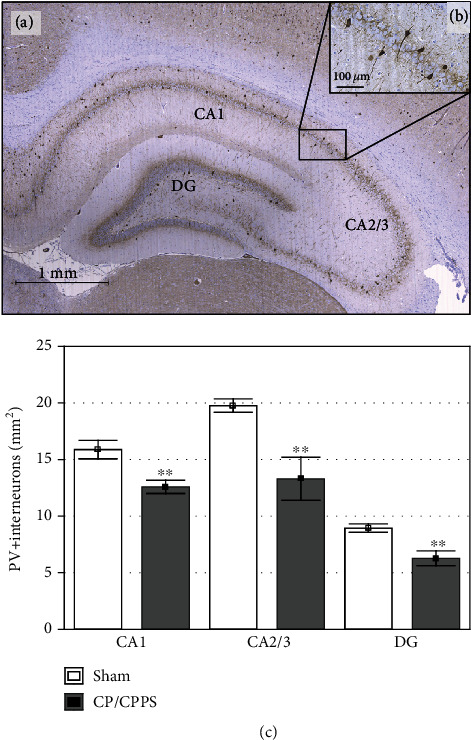
Immunohistochemical expression of PV+ interneurons in the rat hippocampus. Representative image of the rat hippocampus with labelled areas CA1, CA2/3, and DG (a). Scale bar 1 mm. Enlarged image of few PV+ interneurons mainly located in the pyramidal cell layer (b). Scale bar 100 *μ*m. The number of PV+ interneurons in CA1, CA2/3, and DG hippocampal regions (c). Values are mean ± SEM. The statistical significance of the difference between the groups was estimated by Student's *t*-test (^∗∗^*p* < 0.01 vs. sham, *n* = 6 per group). For details, see the caption of Figure 1.

PV+ interneurons in both investigated groups were located mostly within or in the vicinity of a pyramidal cell layer in CA1 and CA2/3 and mostly in the granular cell layer in DG (Figures 9(a) and 9(b)). However, quantitative analysis of PV+ interneuron expression in the three analyzed regions of the hippocampus revealed significant differences between sham and CP/CPPS rats. As shown in Figure 9(c), CP/CPPS animals had significantly less PV+ interneurons in CA1, CA2/3, and DG regions of the hippocampus, compared to sham animals (*p* < 0.01).

### 3.7. Correlation Analysis

**Figure 10:**
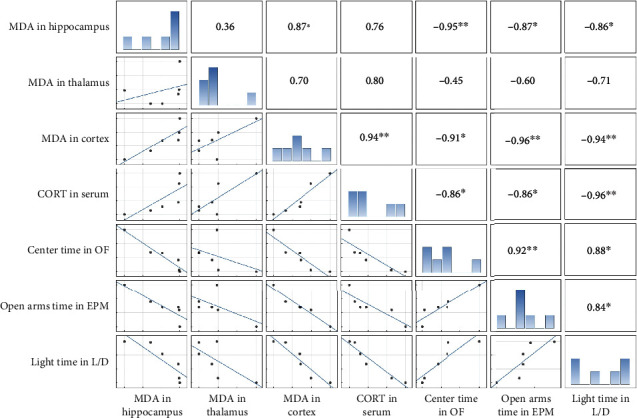
Correlation among parameters of brain oxidative stress, serum corticosterone, and anxiety-like behavior test parameters in CP/CPPS rats. Correlation matrix showing the interrelationship between the MDA levels in the hippocampus, thalamus, and cortex, corticosterone (CORT) levels in serum, time that animal spent in the center of the OF, time that animal spent in the open arms of EPM, and time that animal spent in the light compartment of the L/D box entered into the factor analyses. Histograms showing the distribution of each variable are shown diagonally on the 1 : 1 line. The lower triangular matrix is composed of the bivariate scatter plots with a fitted smooth line, showing the relationships between variables. The upper triangular matrix is composed of Pearson's product-moment correlation that shows the relationship between the variables. The stars show the significant levels of the correlation: ^∗^*p* < 0.05 and ^∗∗^*p* < 0.01, *n* = 6 per group.

A Pearson correlation coefficient was computed to assess the link among the MDA levels in the hippocampus, thalamus, and cortex, as well as serum corticosterone level with parameters of anxiety-like behavior testing in CP/CPPS animals. Results of this correlation analysis is presented as a correlation matrix (Figure 10). Histograms showing the distribution of each variable are positioned diagonally. The lower triangular matrix is composed of the bivariate scatter plots with a fitted smooth line showing the relationships between variables, while the upper triangular matrix is composed of Pearson's correlation coefficients with star-denoted statistical significance showing the type, strength, and statistical significance of the relationship between the variables.

There were strong negative correlations between the MDA level in the hippocampus and the time animal spent in the center of the OF (*r* = −0.95, *p* < 0.01), the time animal spent in the open arms of EPM (*r* = −0.87, *p* < 0.05), and the time animal spent in the light compartment of the L/D box (*r* = −0.86, *p* < 0.05). There was no statistically significant correlation between the MDA level in the thalamus and any analyzed variable. The MDA level in the cortex significantly positively correlated with the corticosterone serum level (*r* = 0.94, *p* < 0.01), while it significantly negatively correlated with the time animal spent in the center of the OF (*r* = −0.91, *p* < 0.05), the time animal spent in the open arms of EPM (*r* = −0.96, *p* < 0.01), and the time animal spent in the light compartment of the L/D box (*r* = −0.94, *p* < 0.01). The corticosterone serum level significantly negatively correlated with the time animal spent in the center of the OF (*r* = −0.86, *p* < 0.05), the time animal spent in the open arms of EPM (*r* = −0.86, *p* < 0.05), and the time animal spent in the light compartment of the L/D box (*r* = −0.96, *p* < 0.01). Also, there were statistically significant correlations among parameters of three different anxiety-like behavior tests (Figure 10).

**Figure 11:**
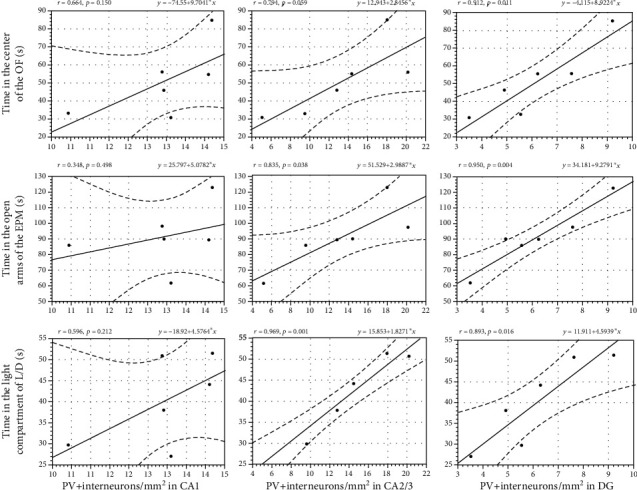
Correlation among the number of PV+ interneurons in different regions of the hippocampus (CA1, CA2/3, and DG) and anxiety-like behavior testing parameters in CP/CPPS rats. Simple regression analysis indicated that the number of PV+ interneurons in the CA2/3 region was positively correlated with parameters of anxiety-like behavior in the EPM and L/D test, as well as that there was a strong positive correlation between the number of PV+ interneurons in DG and parameters of anxiety-like behavior in the OF, EPM, and L/D test. Pearson correlation coefficient (Pearson's *r*) and level of correlation significance (*p* value) are also denoted in the figure, *n* = 6 per group. For details, see the caption of Figure 1.

Also, simple regression analysis was performed to assess the link among the number of PV+ interneurons in CA1, CA2/3, and DG regions of the hippocampus and parameters of anxiety-like behavior testing in the CP/CPPS group. The results of these correlation analyses are presented in Figure 11. Simple regression analysis indicated that the number of PV+ interneurons in CA2/3 was positively correlated with time CP/CPPS animals spent in the open arms of the EPM (*r* = 0.835, *p* < 0.05) and time in the light compartment of the L/D test (*r* = 0.969, *p* < 0.001). Also, there was a strong positive correlation between the number of PV+ interneurons in DG and time these animals spent in the center of the OF test (*r* = 0.912, *p* < 0.05), time in the open arms of the EPM test (*r* = 0.950, *p* < 0.01), and time in the light compartment of the L/D test (*r* = 0.893, *p* < 0.05). There was no significant correlation for the CA1 region and test parameters of anxiety-like behavior (OF, EPM, and LD tests) in CP/CPPS rats (*r* = 0.664, *p* > 0.05; *r* = 0.348, *p* > 0.05; and *r* = 0.596, *p* > 0.05, respectively). The same hold was for the CA2/3 region and time in the center of the OF (*r* = 0.794, *p* > 0.05).

## 4. Discussion

Results of our current study revealed increased anxiety-like behavior in rats with CP/CPPS comparing to corresponding controls, their sham-operated mates. These behavioral changes in CP/CPPS animals were accompanied by increased oxidative stress and alterations in antioxidative capacity in the hippocampus, thalamus, and cerebral cortex, increased serum corticosterone levels, and decreased number of PV+ interneurons in the hippocampus.

We confirmed in our current study the occurrence of CP/CPPS by a functional test, i.e., pain threshold determination, and by histological verification of prostatitis. Namely, NIH-Chronic Prostatitis Symptom Index (NIH-CPSI) showed that chronic pelvic pain and prostatodynia are the most steady and the most important symptoms in patients with CP/CPPS [33]. In our study, CP/CPPS animals showed a statistically significant reduction in mechanical pain threshold in the scrotal skin at 3 and 7 days time points when compared to the sham animals, which is in line with results given in previous studies [26, 27, 34]. Moreover, histological evaluation of prostates from CP/CPPS rats revealed typical pathohistological signs of prostatitis with interstitial leukocyte infiltration and necrosis, cell desquamation, and leukocyte infiltration of tubuloalveolar glands (Figure 3). These outcomes of functional testing and histological image secured face and construct validity of this experimental model, indicating that these time points could be used as a reference point of fully developed CP/CPPS.

### 4.1. Experimentally Induced CP/CPPS Is Accompanied by Anxiety-Like Behavior

Upon induction of CP/CPPS, we have assessed anxiety-like behavior in rats over one week (time points: 2, 3, and 7 days) using a standard battery of ethological tests, including OF, EPM, and L/D. The results of these behavioral tests revealed the appearance of anxiogenic behavior 3 and 7 days upon intraprostatic *λ*-carrageenan injection, while behavioral patterns were unaltered in the first 2 days upon prostatitis induction. Rats with CP/CPPS showed an increased anxiety-like pattern of behavior in the OF, manifested as decreased number of rearings (an indicator of vertical activity), higher index of thigmotaxis (showing its tendency to stick to arena's walls), and lower time spent in the central area, compared to their sham mates (Figure 4). This specific behavioral pattern outlined by these output variables in the OF matches the generally accepted image of the anxiety-like behavior [35]. Also, the remaining two behavioral tests performed herein showed congruent results. Namely, we observed in the EPM decreased time that CP/CPPS animals spent in the open arms and decreased the number of open/closed arm transitions during test 3 and 7 days upon CP/CPPS induction (Figure 5). The EPM is a widely used ethological test for innate anxiety behavior of rodents [36], relying on approach-avoidance behavior easily translational to humans [37]. It is based on rodents' tendency toward dark, enclosed arms (approach) and an unconditioned fear of heights/open arms (avoidance) [38]. In L/D, shorter time spent in the light compartment and decreased number of L/D compartment transitions were scored in CP/CPPS animals in the same time points (Figure 6). The L/D is a widely applied ethological test of unconditioned anxiety-like behavior in rodents, based on conflict between the drive to explore novel areas and an aversion to brightly lit and open areas [39].

Our results are in line with findings from clinical studies indicating the link between CP/CPPS and anxiety, i.e., a higher prevalence of anxiety in men with CP/CPPS [40, 41]. Hence, we confirmed the hypothesis that experimentally induced CP/CPPS is accompanied by anxiety as one of the neuropsychiatric comorbidities, permitting the exploration of the possible mechanism underpinning this link in experimental conditions. Oxidative stress, as an imbalance between ROS production and antioxidative capacities, is one of these possible mechanisms.

### 4.2. Brain Oxidative Stress Could Be the Underlying Mediator of CP/CPPS Link with Anxiety-Like Behavior

Oxidative stress is considered to be a significant factor in the inflammatory cascade of chronic prostatitis development [19, 42]. In the process of any chronic pelvic pain-related syndrome, a large amount of ROS was generated and released into the bloodstream [43, 44]. Moreover, extensive inflammation present in chronic prostatitis leads to stromal or epithelial cell damage [45], thus leading to the intense release of ROS, causing changes in protein structure and function, and DNA modifications [46]. Products of lipid peroxidation, such as MDA and unsaturated aldehydes, are capable of inactivating many cellular proteins. Our results showed that animals with CP/CPPS had significantly increased MDA levels, an indicator of lipid peroxidation, in all examined brain structures, i.e., the hippocampus, thalamus, and cerebral cortex (Figure 7). This suggests a higher oxidative burden in the brains of animals with CP/CPPS. The extent of lipid peroxidation in these structures was almost uniform, indicating the strong trigger of oxidative stress. The brain is very susceptible to oxidative stress damage since it generates more free radicals per gram of tissue than does any other organ, as well as it contains high amount of polyunsaturated fatty acids, high oxygen consumption, and a low antioxidant capacity, compared with other organs [47]. The antioxidant enzymes, including SOD and GPx, as well as content of thiol groups, are among the most efficient mechanisms operating in the brain to tackle the threat posed by ROS [48]. On the other hand, in the current study, there was a significant decrease of SOD activity and thiol group content in all three isolated brain structures in CP/CPPS animals, suggesting significant loss of antioxidants which eventually resulted in the development of oxidative stress. However, CP/CPPS animals had increased activity of GPx enzyme in the thalamus and cortex but not in hippocampal structures, compared to sham animals. We can assume adaptive response in these structures, but still insufficient to cope with oxidative stress in the thalamus and cortex, as well as in the hippocampus.

Neurocircuitry models of anxiety disorders are highly complex and encompass different brain regions and circuits including amygdala-cortical interactions. Cortical regions are believed to be specifically involved in some anxiety disorders [49]. The oxidative stress in the brain could be an underpinning mechanism associated with vulnerability to anxiety disorders [50]. Over the past few years, several studies in rodents reported that anxiety disorders may be characterized by increased oxidative damage to proteins, lipids, and nucleic acids, as well as lowered antioxidant defence [51]. Accumulated oxidative stress could damage neurons and alter neuronal function, resulting in the pathogenesis of anxiety [52]. Also, a link between decreased SOD activity and anxiety was reported [53]. Our results showed that increased anxiety-like behavioral pattern correlated with increased lipid peroxidation in the cortex and hippocampus in CP/CPPS rats. Recently, Bouayed and Soulimani [54] provided direct evidence that hydrogen peroxide, a component of oxidative stress, induces high-anxiety-related behavior in rodents. Further evidence about the role of oxidative stress in the genesis of anxiety has been discussed in more detail elsewhere [51, 55]. Therefore, in our research, we support the hypothesis that oxidative stress in the brain could be the underlying mediator of CP/CPPS link with anxiety-like behavior confirmed herein.

### 4.3. Corticosterone Could Mediate CP/CPPS-Evoked Anxiety-Like Behavior

One of the main features of CP/CPPS, besides prostate inflammation, is chronic pain, and we observed herein marked reduction in pain threshold in CP/CPPS rats. Pain itself is associated with stress and could provoke stress reaction [56]. However, stress, in turn, can be an important causative factor for CP/CPPS and anxiety, independently [9]. Some studies found that high-level stress and lack of social support were associated with a history of prostatitis in men [57].

Results of our study revealed elevated serum corticosterone levels in CP/CPPS rats and its correlation with increased anxiety-like behavior patterns among these rats. This result speaks in favour of increased activity of the HPA axis (CRH-ACTH-corticosterone) and elevated stress level, as well as immune system reactions on stress in CP/CPPS rats. Corticosterone crosses the blood-brain barrier and affects neurons and glial cells producing changes in certain brain regions, such as the prefrontal cortex, amygdala, hippocampus, nucleus accumbens, and hypothalamus [58]. More specifically, corticosterone acts via glucocorticoid and mineralocorticoid receptors in the central nucleus of the amygdala and mediates the HPA and autonomic components of the stress axis [59]. Also, when corticosterone binds at the amygdala, there is an increase in CRH release and the subsequent facilitation of the stress axis since corticosterone might directly affect amygdaloid neurons [60]. Hence, the limbic brain and HPA axis form an interconnected loop as projections from the hippocampus, amygdala, and prefrontal cortex feedback to the hypothalamus and regulate the stress responses and glucocorticoid release [61], together with induction of long-lasting anxiety-like behavior [62]. Recently, Bergamini et al. [63] showed that peripheral pain hypersensitivity increases anxiety by elevating corticosterone serum levels in rats.

Moreover, the decrease in SOD activity in all brain structures described in our study may be due to the release of corticosterone in the response to pain as a stressor. Namely, an earlier study showed that incubation of cortical and hippocampal structures with glucocorticoids resulted in increased ROS accumulation and oxidative stress [64]. We found that elevated serum corticosterone levels positively correlated with lipid peroxidation and oxidative stress in the cortex, thalamus, and hippocampus. It seems that corticosterone, by increasing the availability of glucose and through the Nrf2 signalling pathway, promotes spontaneous ROS generation, increases prooxidant gene transcription, and decreases antioxidant defence mechanisms [65]. Also, excessive ROS production leads to dysregulation of inflammatory response and degradation of NF-*κ*B inhibitors [66]. Therefore, our current results support the hypothesis that activation of the HPA-corticosterone axis could be another underlying mediator of CP/CPPS link with anxiety-like behavior together with oxidative stress.

### 4.4. Hippocampal PV+ Interneuron Reduction in CPPS-Evoked Anxiety-Like Behavior

Numerous studies suggested an important role of the hippocampus and its alterations in anxiety initiation and development [67–69]. Anxiety is the result of competition between simultaneously available goals or choices that could be associated with conflict or uncertainty [70]. Different hippocampal neural circuits are considered to be the main part of a comparator system to detect it [71]. The hippocampus is organized in the stereotyped anatomical trisynaptic circuit [72]. Cortical inputs from the entorhinal cortex carry spatial and contextual information, synapsing onto dentate gyrus (DG) granule cells and area CA3 pyramidal neurons, which in turn project to area CA2 and CA1 pyramidal neurons that send extrahippocampal projections [73]. Highly interconnected reciprocal circuit, composed of extrahippocampal projections, amygdala, medial prefrontal cortex, hypothalamus, and the bed nucleus of the stria terminals [74] can elicit anxiety-related behavior via direct outputs to brainstem structures like the periaqueductal grey and parabrachial nucleus [75]. Hippocampal PV+ interneurons, predominantly chandelier (or axo-axonic) cells, and a subset of basket-type interneurons [24] are involved in the regulation of cognitive function, circadian rhythms, and behavioral patterns including anxiety, social interaction, and fear extinction [67]. These interneurons provide inhibition in the cortex and hippocampus by control of the memory-related network activity pattern generation which is involved in the obtaining and extinction of fear memories [76]. The main suppressive effect of hippocampal PV+ interneurons on hippocampal CA1, CA 2/3, and DG neuronal activity is mediated mainly by GABAergic neurotransmission [77].

Results of our study showed decreased number of PV+ interneurons in CA1, CA2/3, and DG regions of the hippocampus in CP/CPPS rats. Moreover, this decreased number of hippocampal PV+ interneurons in CA2/3 and DG regions correlated with increased anxiety-like behavioral patterns during OF, EPM, and L/D tests in CP/CPPS rats. The simple and unconditioned ethological laboratory tests of anxiety are based on the conflict between the approach and exploration of the potential danger, but also potential reward (open arm in EPM, the center of the OFT, and light compartment of the L/D test), or avoidance in the safe and enclosed compartments (closed arm in EPM, the periphery of the OFT, and dark compartment of the L/D test) [71]. Taking into account these facts, it could explain why the impaired hippocampal PV+ interneuronal network caused anxiety-like behavior, which even correlated with the degree of PV+ interneuron loss.

There are many speculated mechanisms implicated in alterations of hippocampal plasticity in CP/CPPS rats. Impaired redox status in the brain is observed to be responsible for many immunohistochemical changes in the hippocampus [78]. Namely, redox dysregulation impairs glutamatergic, dopaminergic, immune, and antioxidant signalling in the brain [25], as well as NADPH oxygenase 2 regulation [79], which is causally connected to PV+ interneuron loss. Additionally, corticosterone could generate a considerable reduction in the hippocampal PV+ interneuron expression. Exposure to chronic stress and recruitment of HPA axis with elevated serum level of corticosterone may lead to dendritic retraction without cell death thus compromising hippocampal function [80]. The hippocampus acts as a negative feedback control to the HPA axis by downregulation of CRH production in the hypothalamus [81]. The loss of hippocampal-negative feedback regulation of the HPA axis may be associated with elevated serum corticosterone levels in CP/CPPS rats, which could in turn induce hippocampal changes. Therefore, loss of hippocampal PV+ interneurons is responsible for further HPA axis hyperactivity in the positive-feedback loop manner [82], making CP/CPPS animals more vulnerable to chronic-stress-induced anxiety-like behavioral alterations. Moreover, chronic pain could induce hippocampal maladaptive plastic changes by decreasing hippocampal dendritic complexity and reducing the number of PV+ interneurons [21]. Therefore, our findings indicated the involvement of hippocampal PV+ interneuron reduction in CP/CPPS link with anxiety-like behavior.

### 4.5. The Interplay between Brain Oxidative Stress, Serum Corticosterone, and Hippocampal PV+ Interneurons in CP/CPPS-Evoked Anxiety-Like Behavior

Anxiety presents primarily a response to the potential danger and could be a protective mechanism to fall into potentially dangerous situations [83], which is used as a base of ethological tests to assess anxiety-like behavior in laboratory animals. Herein, there were strong positive correlations among parameters of three different anxiety-like behavior tests (OF, EPM, and L/D) used in this study, all indicating increased anxiety-like behavior in CP/CPPS rats. On the other hand, we have demonstrated that increased anxiety-like behavior significantly correlated with increased lipid peroxidation in the cortex and hippocampus in CP/CPPS rats measured as the MDA level in these brain structures. Furthermore, elevated serum corticosterone level positively correlated with indicators of anxiety-like behavior but also with lipid peroxidation in the cortex in CP/CPPS rats. We also demonstrated that there was a strong correlation between increased anxiety-like behavior and number of hippocampal PV+ interneurons in CA2/3 and DG regions in CP/CPPS rats. Namely, a lower number of PV+ interneurons in CA2/3 was accompanied by less time CP/CPPS animals spent in the open arms of the EPM and less time in the light compartment of the L/D test. Similar situation existed for PV+ interneurons in DG. Hence, an interplay between brain oxidative stress, hyperactivity of HPA axis seen as elevated serum corticosterone, and loss of hippocampal PV+ interneurons exist in CP/CPPS-evoked anxiety-like behavioral patterns with possible involvement of *circulus vitiosus* mechanisms.

## 5. Conclusions

In summary, our current study showed increased anxiety-like behavior in rats with CP/CPPS which strongly correlated with increased oxidative stress in the brain, elevated corticosterone serum level, and hippocampal PV+ interneurons loss. These mechanisms could be potential targets for the amelioration of psychiatric comorbidities in CP/CPPS. Also, we cannot exclude other possible mechanisms of anxiety-like behavior in CP/CPPS, which remains to be elucidated in further studies.

## Data Availability

The data used to support the findings of this study are available from the corresponding author upon request.
